# Postoperative infections after robotic‐assisted radical prostatectomy in a single large institution: Effect of type and duration of prophylactic antibiotic administration

**DOI:** 10.1111/iju.15635

**Published:** 2024-11-18

**Authors:** Masao Mitsui, Takuya Sadahira, Naoya Nagasaki, Yuki Maruyama, Takanori Sekito, Takehiro Iwata, Satoshi Katayama, Kensuke Bekku, Motoo Araki

**Affiliations:** ^1^ Department of Urology Okayama University Graduate School of Medicine, Dentistry and Pharmaceutical Sciences Kita‐ku Okayama Japan

**Keywords:** cefazolin, postoperative infections, prophylactic antibiotics, prostate, robotic‐assisted radical prostatectomy

## Abstract

**Objective:**

We evaluated the incidence of and risk factors for postoperative infections after robotic‐assisted radical prostatectomy (RARP) according to the type and duration of prophylactic antibiotic administration.

**Methods:**

A total of 1038 patients underwent RARP at our institution from 2010 to 2021; 1026 patients (201 in the cefazolin [CEZ] group and 825 in the ampicillin/sulbactam [ABPC/SBT] group) were analyzed, and 12 who used other antibiotics were excluded. The primary endpoint was the incidence of urinary tract infection (UTI), surgical site infection (SSI), and remote infection (RI). T‐tests, propensity score matching (PSM) and inverse probability of treatment weighting (IPTW) were performed. Multivariate logistic regression analysis was performed to evaluate the effect of type and duration of prophylactic antibiotic administration.

**Results:**

The incidence of UTI was 2.5% (5/201) in the CEZ group and 3.2% (26/825) in the ABPC/SBT group, with no significant difference between groups (*p* = 0.622). The rates of SSI and RI were comparable between groups (*p* = 0.680 and 0.906, respectively). Although the duration of antimicrobial therapy was longer in the ABPC/SBT group (*p* < 0.001), there was no significant difference in the incidence of UTI/SSI/RI after PSM and IPTW (all *p* > 0.05). Multivariate logistic regression analysis showed that neither the type of antibiotic nor the duration of administration affected the incidence of UTI/SSI/RI.

**Conclusion:**

The risk of postoperative UTI/SSI/RI after RARP did not change with the type and duration of antimicrobial therapy.

**Abbreviations & Acronyms**
ABPC/SBT: ampicillin/sulbactam
CEZ: cefazolin
IPTW: inverse probability of treatment weighting
PSM: propensity score matching
RARP: robotic‐assisted radical prostatectomy
RI: remote infection
SSI: surgical site infection
UTI: urinary tract infection

## INTRODUCTION

Robotic‐assisted radical prostatectomy (RARP) is classified as a clean‐contaminated operation because it is not necessarily sterile.^1^, ^2^ Therefore, prophylactic antibiotic administration is important for the prevention of postoperative infections.^3^ The recommended prophylactic antimicrobial agent is a first‐generation cephalosporin according to the Centers for Disease Control and Prevention (CDC) guidelines^1^, ^2^ and a first‐generation cephalosporin or penicillin in combination with a beta‐lactamase inhibitor according to the Japanese Urological Association (JUA) and the American Urological Association (AUA) guidelines.^4^, ^5^ Many studies have compared the incidence of postoperative infections by the duration of antimicrobial administration,^6^, ^7^ but few have compared the incidence by the type of prophylactic antimicrobial agent administered. In this study, we evaluated the incidence of postoperative infections after RARP according to the type of administered antimicrobial agent and the duration of administration.

## MATERIALS AND METHODS

### Patients

**FIGURE 1:**
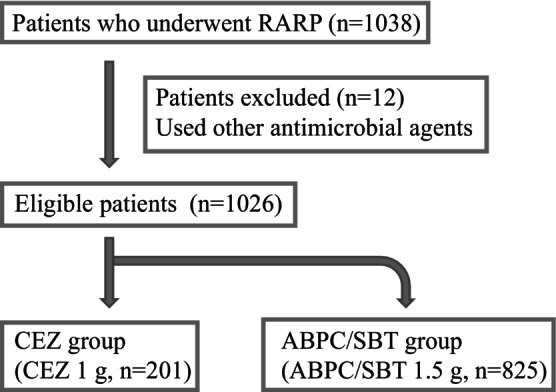
Trial profile and number of patients classified into the 2 groups. Of the 1038 patients, 201 patients who used CEZ and 825 patient who used ABPC/SBT. We excluded 12 patients who used antimicrobial agents other than CEZ or ABPC/SBT.

We examined 1038 patients with prostate cancer who underwent RARP at Okayama University Hospital between October 2010 and August 2021. Patients were classified into two groups: 201 patients who used cefazolin (CEZ), a first‐generation cephalosporin, and 825 patients who used ampicillin/sulbactam (ABPC/SBT), penicillin in combination with a beta‐lactamase inhibitor, as prophylactic antimicrobial therapy. Patients with a history of urinary tract infection (UTI) underwent urine culture. Twelve patients who used other antimicrobial agent because of the detection of resistant bacteria or the history of allergy were excluded (Figure 1).

### Surgical technique and postoperative management

More than 10 surgeons performed the procedures. In most cases, we used a transperitoneal approach and did not spare the Retzius space. After prostate removal, a vesicourethral anastomosis was performed, and a closed suction drain was placed in the pelvic floor to complete the procedure. On the sixth postoperative day, cystography was performed, and if leakage was found at the vesicourethral anastomosis, the urethral catheter was kept in place, and at a later date, cystography was performed again.

### Selection and administration of antimicrobial agents

Administration of prophylactic antimicrobial agents was not randomized, because preoperative bacteriuria‐positive cases were observed in eligible patients. These cases were treated with antimicrobial agents by the day before surgery. The choice of prophylactic antimicrobial agents and the duration of administration were determined at the attending physician's discretion based on the JUA and the AUA guidelines.^4^, ^5^ As a rule, CEZ 1 g or ABPC/SBT 1.5 g was selected. Prophylactic antimicrobial agents were intravenously administered immediately before surgery, with additional doses given every 4 h during surgery. In principle, antimicrobial agents were not changed except in cases of febrile infections.

### Study design and statistical analysis

Patient background characteristics and incidence of postoperative infections between groups were compared, and the influence of antimicrobial type (CEZ, ABPC/SBT) and duration of administration (within 24 h, beyond 24 h) were examined. The primary endpoint was the incidence of urinary tract infection (UTI), surgical site infection (SSI), and remote infection (RI) within 30 days after surgery. UTI was defined as fever with 10^5^ CFU/mL of microorganisms in urine with fever, costovertebral angle tenderness, or scrotal pain. SSI was defined according to the CDC criteria.^1^, ^2^ RI was organ‐specific. For example, pneumonia was defined as the presence of any three criteria of positive bacteriology: fever, purulent sputum, rales, increased white blood cell (WBC) count, and positive X‐ray findings. Clinical comparisons were done by chi‐squared test, Fisher's exact test, and group T‐test. Propensity score matching (PSM) and inverse probability of treatment weighting (IPTW) were performed to adjust for patient background characteristics that may influence the effect of prophylactic antimicrobial agents. Propensity scores were calculated using a logistic regression model including age, body mass index (BMI), initial prostate‐specific antigen (PSA), glucose intolerance, use of steroids or immunosuppressive drugs, clinical stage, preoperative bacteriuria, operation time, bleeding amount, lymph node dissection, postoperative anastomotic leak, duration of antimicrobial therapy and duration of urinary catheter placement. The patients in the two groups were matched in a 1:1 ratio according to the propensity score. In IPTW, propensity scores were calculated using a logistic regression model including age, BMI ≥30, hemoglobin A1c (HbA1c) ≥7, initial PSA, use of steroids or immunosuppressive drugs, clinical stage, and preoperative pyuria. Multivariate logistic regression analysis was performed to evaluate the impact of antimicrobial type (CEZ, ABPC/SBT) and duration of administration (within 24 h, beyond 24 h) on the risk of postoperative infection. SPSS ver. 29.0 was used for statistical analysis, with *p* < 0.05 for statistical significance.

## RESULTS

### Clinicopathological characteristics of patients and the incidence of postoperative infections

There were 201 patients in the CEZ group and 825 in the ABPC/SBT group (Figure 1). The CEZ group was older, required less lymph node dissection, had more cT3 or more, and had a longer operation time than the ABPC/SBT group (Table 1). The ABPC/SBT group included many initial cases in the observation period. The duration of antibiotic administration was longer in the ABPC/SBT group (*p* < 0.001). Catheterization period beyond 7 days were 7.1% (11/154) within 24 h and 19.1% (9/47) beyond 24 h in the CEZ group, and 15.0% (66/441) within 24 h and 10.7% (41/384) beyond 24 h in the ABPC/SBT group. The incidence of postoperative infections (Table 2) was 5.0% (10/201) in the CEZ group and 5.7% (47/825) in the ABPC/SBT group, with no significant difference between groups (*p* = 0.689). The incidence of UTI was 2.5% (5/201) in the CEZ group and 3.2% (26/825) in the ABPC/SBT group, with no significant difference between groups (*p* = 0.622). In addition, the incidences of SSI and RI were not significantly different between groups (*p* = 0.680 and 0.906, respectively). In the ABPC/SBT group, the incidences of UTI was lower in the duration of antimicrobial therapy beyond 24 h than within 24 h (*p* = 0.015) (Table 3). The type of postoperative infections in the CEZ group included 5 patients who experienced UTI (2 epididymitis, 3 pyelonephritis), 4 SSI (2 superficial SSI, 2 deep/organ space SSI), and 2 RI (pneumonia); in the ABPC/SBT group, there were 26 UTI (2 epididymitis and 24 pyelonephritis), 13 SSI (9 superficial SSI and 4 deep/organ‐space SSI), and 9 RI (3 upper respiratory tract infection, 4 pneumonia, 1 intra‐abdominal infection, and 1 other).

**Table 1:** Clinical characteristics of the 1026 patients before and after propensity score matching.

Characteristic	Unmatched			Matched		
	CEZ	ABPC/SBT	*p* value	CEZ	ABPC/SBT	*p* value
	*n* = 201	*n* = 825		*n* = 191	*n* = 191	
Age, years, median (IQR)	68.9 (65–72.5)	67.5 (64–72)	0.002	68.6 (65.0–72.5)	68.7 (65.0–72.0)	0.964
BMI, kg/m^2^, median (IQR)	24.0 (22.2–25.7)	23.8 (21.9–25.4)	0.236	24.0 (22.5–25.3)	24.0 (22.2–25.6)	0.937
Initial PSA, ng/mL, median (IQR)	9.97 (5.39–11.8)	10.44 (5.42–11.1)	0.088	11.0 (5.32–11.2)	9.78 (5.43–11.7)	0.196
HbA1c ≧7%, *n* (%)	10 (5.0)	34 (4.1)	0.592	10 (5.4)	4 (2.1)	0.173
Use of steroids or immunosuppressive drug, *n* (%)	2 (1.0)	9 (1.1)	0.100	6 (3.1)	1 (0.5)	0.122
Staging, *n* (%)			<0.001			0.459
≤cT2	171 (85.1)	766 (92.8)		162 (84.8)	167 (87.4)	
≥cT3	30 (14.9)	59 (7.2)		29 (15.2)	24 (12.6)	
Preoperative pyuria, *n* (%)	3 (1.5)	14 (1.7)	1.00	3 (1.6)	2 (1.0)	1.00
Operation time, min, median (IQR)	225.3 (191–250)	212.7 (175–241)	0.006	222 (181–254)	226 (191–253)	0.59
Estimated blood loss, mL, median (IQR)	173.7 (50–200)	161.4 (50–200)	0.494	165.4 (50–200)	179 (50–200)	0.557
Lymph node dissection, *n* (%)	108 (53.7)	562 (68.1)	<0.001	110 (57.6)	106 (55.5)	0.680
Intestinal injury, *n* (%)	1 (0.5)	1 (0.1)	0.354	1 (0.5)	0 (0)	1.00
Prophylactic antibiotic administration period, *n* (%)			<0.001			0.904
≤24 h	154 (76.6)	441 (53.5)		146 (76.4)	145 (75.9)	
>24 h	47 (23.4)	384 (46.5)		45 (23.6)	46 (24.1)	
Post‐operative urinary leak, *n* (%)	8 (4.0)	50 (6.1)	0.252	8 (4.2)	8 (4.2)	1
Catheterization period >7 days, *n* (%)	20 (10.0)	107 (13.0)	0.244	16 (8.4)	19 (9.9)	0.595

Abbreviations: ABPC/SBT, ampicillin/sulbactam; BMI, body mass index; CEZ, cefazolin; IQR, interquartile range; PSA, prostate‐specific antigen.

**Table 2:** Incidence of postoperative infections before and after propensity score matching.

Characteristic	Unmatched			Matched		
	CEZ	ABPC/SBT	*p* value	CEZ	ABPC/SBT	*p* value
	*n* = 201	*n* = 825		*n* = 191	*n* = 191	
Postoperative infection, *n* (%)	10 (5.0)	47 (5.7)	0.689	9 (4.1)	7 (3.7)	0.609
Urinary tract infection (UTI)	5 (2.5)	26 (3.2)	0.622	4 (2.1)	3 (1.6)	0.703
Surgical site infection (SSI)	4 (2.0)	13 (1.6)	0.680	4 (2.1)	2 (1.0)	0.411
Remote infection (RI)	2 (1.1)	9 (1.1)	0.906	1 (0.5)	2 (1.0)	0.562

Abbreviations: ABPC/SBT, ampicillin/sulbactam; CEZ, cefazolin.

**Table 3:** Incidence of postoperative infections before propensity score matching: within 24 h and beyond 24 h.

Characteristic	CEZ (*n* = 201)			ABPC/SBT (*n* = 825)		
	≤24 h	>24 h	*p* value	≤24 h	>24 h	*p* value
	*n* = 154	*n* = 47		*n* = 441	*n* = 384	
Postoperative infection, *n* (%)	9 (5.8)	1 (2.1)	0.305	30 (6.8)	17 (4.4)	0.142
Urinary tract infection (UTI)	4 (2.6)	1 (2.1)	0.856	20 (4.5)	6 (1.6)	0.015
Surgical site infection (SSI)	4 (2.6)	0 (0.0)	0.264	5 (1.1)	8 (2.1)	0.275
Remote infection (RI)	2 (1.3)	0 (0.0)	0.432	5 (1.1)	4 (1.0)	0.899

Abbreviations: ABPC/SBT, ampicillin/sulbactam; CEZ, cefazolin.

### Comparing and contrasting after PSM and IPTW, multivariate logistic regression analysis

A total of 191 cases in each group were selected by propensity score matching (Table 1). There were no significant differences in clinical characteristics between groups. The incidence of postoperative infections was 4.1% (9/191) in the CEZ group and 3.7% (7/191) in the ABPC/SBT group, with no significant difference between groups (*p* = 0.609) (Table 2). In addition, the incidences of SSI/UTI/RI were not significantly different between groups (Tables 2 and 4). IPTW had similar results (all *p* > 0.05) (Table 5). Multivariate logistic regression analysis showed that a longer duration of catheterization significantly increased the risk of postoperative infections (OR 6.56, 95% CI 3.72–11.6, *p* < 0. 01). The type and duration of prophylactic antibiotic administration were not risk factors for infection (OR 1.13, 95% CI 0.541–2.73, *p* = 0.741, and OR 0.625, 95%CI 0.339–1.15, *p* = 0.132, respectively) (Table 6).

**Table 4:** Incidence of postoperative infections after propensity score matching: within 24 h and beyond 24 h.

Characteristic	CEZ (*n* = 191)			ABPC/SBT (*n* = 191)		
	≤24 h	>24 h	*p* value	≤24 h	>24 h	*p* value
	*n* = 145	*n* = 46		*n* = 146	*n* = 45	
Postoperative infection, *n* (%)	8 (5.5)	1 (2.2)	0.351	7 (4.8)	0 (0.0)	0.135
Urinary tract infection (UTI)	3 (2.1)	1 (2.2)	0.966	3 (2.1)	0 (0.0)	0.332
Surgical site infection (SSI)	4 (2.8)	0 (0.0)	0.255	2 (1.4)	0 (0.0)	0.430
Remote infection (RI)	1 (0.7)	0 (0.0)	0.572	2 (1.4)	0 (0.0)	0.430

Abbreviations: ABPC/SBT, ampicillin/sulbactam; CEZ, cefazolin.

**Table 5:** Effect of CEZ on the risk of post‐operative infections after IPTW (vs. ABPC/SBT).

	Odds ratio	95% CI	*p* value
Post‐operative infection	1.319	0.602, 2.893	0.489
Urinary tract infection (UTI)	0.396	0.148, 1.057	0.064
Surgical site infection (SSI)	1.632	0.491, 5.425	0.424
Remote infection (RI)	0.738	0.142, 3.832	0.717

Abbreviations: ABPC/SBT, ampicillin/sulbactam; CEZ, cefazolin; CI, confidence interval; IPTW, inverse probability of treatment weighting.

**Table 6:** Multivariate logistic regression analysis of risk factors for postoperative infections (UTI/SSI/RI).

	Odds ratio	95% CI	*p* value
Age	0.998	0.953, 1.040	0.933
HbA1c ≧7	1.700	0.563, 5.150	0.345
Preoperative pyuria	2.01	0.406, 9.930	0.392
Lymph node dissection	0.933	0.502, 1.730	0.826
Catheterization period >7 days	6.560	3.720, 11.60	<0.01
Use of ABPC/SBT	1.130	0.541, 2.730	0.741
Postoperative antibiotic administration period >24 h	0.625	0.339, 1.150	0.132

Abbreviations: ABPC/SBT, ampicillin/sulbactam; CI, confidence interval; RI, remote infection; SSI, surgical site infection; UTI, urinary tract infection.

## DISCUSSION

In this study, the incidence of postoperative infections was not significantly different between the CEZ group (5.0%) and the ABPC/SBT group (5.7%) (*p* = 0.689). We used PSM and IPTW to minimize imbalance, and we confirmed that there was no significant difference in the incidence of postoperative infections between the CEZ group and the ABPC/SBT group (all *p* > 0.05). CEZ was shown to be potentially as effective as ABPC/SBT in preventing postoperative infections for RARP. Further, multivariate logistic regression analysis showed that neither the type of antimicrobial agent (CEZ, ABPC/SBT) nor the duration of administration (within 24 h or beyond 24 h) affected the incidence of postoperative infections. The results indicated that prophylactic antimicrobial agents may be effective in preventing postoperative infections by a method of administration limited to 24 h of CEZ if antibiotic‐resistant bacteria are not detected.

RARP is classified as a clean‐contaminated operation.^1^, ^2^ Although prophylactic antimicrobial agents may not be necessary for a clean operation,^1^, ^8^ they are considered important in the prevention of postoperative infections for clean‐contaminated operations.^3^, ^9^ Many previous studies of radical prostatectomy compared the incidence of postoperative infection by the duration of prophylactic antimicrobial administration,^6^, ^7^ but few compared the incidence by type of antibiotic. Due to a lack of evidence, prophylactic antimicrobial agents are similar to those used in clean‐contaminated operations involving other organs. In previous studies, CEZ was shown to be useful as a prophylactic antimicrobial agent in clean‐contaminated operations in gynecology,^10^ gastrointestinal surgery,^11^ and head and neck surgery.^12^, ^13^ In this study, we evaluated the prophylactic antimicrobial agents for RARP in a clean‐contaminated operation, and CEZ was shown to be useful. Our study further supports existing guidelines.^1^, ^2^, ^4^, ^5^

Prophylactic antimicrobial agents should be selected based on microbial susceptibility, resistance, and transition of the agent into the tissue covering the suspected contaminating bacterial species in each target organ. Currently, the incidence of drug‐resistant bacteria is increasing. Therefore, we must use antimicrobial agents appropriately and use those with the narrowest possible spectra. CEZ, a relatively narrow‐spectrum antibacterial agent, is effective against methicillin‐sensitive *Staphylococcus aureus* (MSSA), *Streptococcus* spp., *Escherichia coli*, *Klebsiella pneumoniae*, and *Proteus mirabilis*. In addition to these bacteria, the use of ABPC/SBT is also effective against *Enterococcus faecalis* and anaerobic bacteria. Because they are both active against the suspected contaminating bacterial species in skin and urinary tract and migrate to the urinary tract as well as the skin,^14^, ^15^ they are reasonable prophylactic antimicrobial agents to use for the prevention of SSI/UTI after prostatectomy. However, most guidelines focus only on SSIs, and the efficacy for RI has not been well‐studied. For example, a representative RI is pneumonia, which is caused by aspiration of anaerobic bacteria in the oral cavity. In this study, there was no significant difference between the ABPC/SBT group, which covers anaerobic bacteria, and the CEZ group, which does not, suggesting that CEZ and ABPC/SBT may have the comparable RI suppression effect.

The incidence of postoperative infections varies among previous reports. The incidence of SSI, including infectious lymphocele, generally ranged from 0.4% to 2.0%,^16^, ^17^, ^18^, ^19^ and the incidence of UTI, including epididymitis, ranged from 0.2% to 3.3%^18^, ^20^ after RARP. In this study, the incidence of SSI/UTI did not differ significantly between groups, and it was within the range of other reports. With regard to SSI, the frequency of bowel injury was similar to other reports,^16^ and lymph node dissection was performed more often compared with other reports.^18^ Lymph node dissection was reported to increase the incidence of infectious lymphocele,^21^ but multivariate logistic regression analysis showed that lymph node dissection was not a risk factor for infection in this study. In most cases, lymph node dissection was performed in a localized area, which may have had less impact on SSI. If there was a possibility of infection, it may have been related to a lack of disinfection of the operative field with alcohol‐containing products, double glove use, and changing gloves every 3 h, as recommended by the CDC guidelines.^1^, ^2^ In this study, both groups of patients were treated under the same conditions, so those issues did not affect the analysis. However, we believe that it is necessary to improve SSI prevention measures in accordance with the guidelines. With regard to UTI, cystography was performed in all cases, and if there was leakage at the anastomosis, the urethral catheter was left in place. While anastomotic leakage could be diagnosed quickly, it inevitably prolonged the catheterization period. A prolonged catheterization period was reported to increase the incidence of postoperative infections,^6^ which may have led to an increase in UTI. The main reason for a prolonged catheterization period was anastomotic leakage, suggesting that it is necessary to reduce postoperative anastomotic leakage and that a more secure vesicourethral anastomosis is important in the prevention of postoperative infections.

In the ABPC/SBT group, the duration of antimicrobial therapy was longer (*p* < 0.001), and the incidences of UTI was lower in the duration of antimicrobial therapy beyond 24 h (*p* = 0.015) (Table 3). This was because the ABPC/SBT group included many initial cases in the observation period, and we may have tended to administer ABPC/SBT, a broad‐spectrum antibacterial agent, for longer periods of time. And, catheterization period tended to be shorter in the duration of antimicrobial therapy beyond 24 h. The duration of antimicrobial administration could well have affected the incidence of postoperative infections, so we used PSM, IPTW, and multivariate logistic regression analysis. In IPTW, the incidences of UTI was insignificantly lower in the CEZ group (OR 0.396, 95% CI 0.148–1.057, *p* = 0.064). This was because postoperative anastomotic leakage was less and catheterization period was shorter in the CEZ group. IPTW was selected only preoperative factors for propensity scores and did not include postoperative factors. They showed that neither the type of antimicrobial agent administered (CEZ, ABPC/SBT) nor the duration of administration (within 24 h, beyond 24 h) affected the incidence of postoperative infections. The results indicated that prophylactic antimicrobial agents may be effective in preventing postoperative infections by a method of administration limited to 24 h of CEZ, if antibiotic‐resistant bacteria are not detected.

Currently, the increasing incidence of drug‐resistant bacteria and rising medical costs have become major issues. We must avoid the overuse of broad‐spectrum antimicrobial agents and provide the best possible medical care to our patients at a limited cost. If CEZ is sufficient to prevent postoperative infections as a prophylactic antimicrobial agent, we may reduce the frequent use of broad‐spectrum ABPC/SBT and help prevent the development of drug resistance in bacteria. In addition, CEZ is less expensive than ABPC/SBT, which may lead to a reduction in medical costs. In the future, it is desirable to re‐evaluate the economics of drug therapy based on all factors related to drug therapy (e.g., duration of administration, treatment of perioperative infections, treatment of side effects, etc.), not just a direct comparison of drug prices.

Limitations of this study include its retrospective, single‐center design; thus, randomization of antimicrobial agents was not possible, and adjustment for unmeasured confounders such as the initial and last cases could not be carried out because PSM was used. In the future, a multicenter, randomized, controlled trial should be performed to confirm our findings.

In conclusion, the risk of UTI/SSI/RI after RARP did not change with the type and duration of antimicrobial therapy. Prophylactic antimicrobial agents may be effective in preventing postoperative infections by a method of administration limited to 24 h of CEZ.

## AUTHOR CONTRIBUTIONS

**Masao Mitsui:** Writing – original draft; writing – review and editing. **Takuya Sadahira:** Conceptualization; supervision. **Naoya Nagasaki:** Data curation; investigation; formal analysis. **Yuki Maruyama:** Supervision. **Takanori Sekito:** Supervision. **Takehiro Iwata:** Supervision. **Satoshi Katayama:** Supervision. **Kensuke Bekku:** Supervision. **Motoo Araki:** Supervision.

## CONFLICT OF INTEREST STATEMENT

Motoo Araki is an Editorial Board member of International Journal of Urology and a co‐author of this article. To minimize bias, they were excluded from all editorial decision‐making related to the acceptance of this article for publication.

## APPROVAL OF THE RESEARCH PROTOCOL BY AN INSTITUTIONAL REVIEWER BOARD

Approval number: K2011‐015.

## INFORMED CONSENT

Written consent was waived because of the study's retrospective nature, and patients provided consent via an opt‐out approach.

## REGISTRY AND THE REGISTRATION NO. OF THE STUDY/TRIAL

N/A.

## ANIMAL STUDIES

N/A.
